# Xylitol-Containing Chewing Gum Reduces Cariogenic and Periodontopathic Bacteria in Dental Plaque—Microbiome Investigation

**DOI:** 10.3389/fnut.2022.882636

**Published:** 2022-05-11

**Authors:** Yi-Fan Wu, Eisner Salamanca, I-Wen Chen, Jo-Ning Su, Yu-Che Chen, Sin Yu Wang, Ying-Sui Sun, Nai-Chia Teng, Wei-Jen Chang

**Affiliations:** ^1^School of Dentistry, College of Oral Medicine, Taipei Medical University, Taipei, Taiwan; ^2^School of Dental Technology, Taipei Medical University, Taipei, Taiwan; ^3^Division of Oral Rehabilitation and Center of Pediatric Dentistry, Department of Dentistry, Taipei Medical University Hospital, Taipei, Taiwan; ^4^Dental Department, Taipei Medical University-Shuang Ho Hospital, New Taipei City, Taiwan

**Keywords:** oral microbiota, oral health and hygiene, dental plaque, dental caries (DMFT), 16S rDNA sequencing analysis, xylitol gum, periodontal health, periodontopathic bacteria

## Abstract

**Background:**

Dental caries and periodontal disease remain the most prevalent oral health problems in the world. Chewing xylitol gum may help reduce the risk of caries and periodontitis for dental health benefits. However, little evidence has shown healthy food estimation by sequencing 16S rDNA in oral microbial communities. This study investigated the clinical effect of xylitol chewing gum on dental plaque accumulation and microbiota composition using the PacBio full-length sequencing platform in 24 young adults (*N* = 24). The participants were randomly assigned to xylitol chewing gum and control (no chewing gum) groups. Participants in the chewing gum group chewed ten pieces of gum (a total of 6.2 g xylitol/day). Dental plaque from all teeth was collected for weighing, measuring the pH value, and analysis of microbial communities at the beginning (baseline, M0) and end of the 2-week (effect, M1) study period.

**Results:**

The results suggested a 20% reduction in dental plaque accumulation (*p* < 0.05) among participants chewing xylitol gum for 2 weeks, and the relative abundance of *Firmicutes* (a type of pathogenic bacteria associated with caries) decreased by 10.26% (*p* < 0.05) and that of *Bacteroidetes* and *Actinobacteria* (two types of pathogenic bacteria associated with periodontitis) decreased by 6.32% (*p* < 0.001) and 1.66% (*p* < 0.05), respectively. Moreover, the relative abundance of *Fusobacteria* was increased by 9.24% (*p* < 0.001), which has been proven to have a higher proportion in dental plaque of healthy adults. However, the dental plaque pH value stayed in a healthy range for the two groups.

**Conclusion:**

In conclusion, chewing xylitol gum would benefit cariogenic and periodontal bacterial reduction in the oral cavity, which could help to prevent the diseases related to these bacteria.

## Introduction

Dental caries and periodontal disease are the most prevalent oral problems in humans, and each one alone can lead to tooth loss. The World Health Organization (WHO) has indicated that almost all adults and over 60% of children have dental caries (1). The occurrence of caries from clinical research is higher in patients with diets of high carbohydrate intake and lower saliva production, and cariogenic microorganisms adhere to the biofilm on the tooth surface. These factors can process hard tissue minerals over time and destroy their organic components (2). For example, *Streptococcus* is considered a highly cariogenic bacterium capable of this destruction (3, 4). Many assessments can evaluate the oral health status, such as the weight of dental plaque (5), microbial community (6), pH value of dental plaque (7), and colonization measurement of specific cariogenic bacteria (8, 9).

Xylitol is a 5-carbon sucrose substitution (C_2_H_12_O_5_, molecular weight: 152.15 g/mol) derived from beechwood, also found naturally in small amounts inside strawberries and cauliflowers (10). This natural sugar is accepted as a safe substance to reduce dental caries risks (11). Xylitol can disrupt energy production process (EPS) of cariogenic microorganisms and decrease acid production from bacterial fermentation and bacterial adherence ability on teeth surfaces (12). Chewing gum is one of the most ubiquitous products around the globe, with a world market fabrication of more than 560,000 tons annually (13). Xylitol gum has been proven to have many advantages, such as stimulating saliva flow, increasing the pH value, and eliminating food debris and dental plaque easily (14, 15). In the last decades, the Food and Drug Administration (FDA) and European Food Safety Authority (EFSA) have approved Xylitol consumption as part of a balanced diet (16, 17). Different researchers have studied the inhibition of cariogenic bacteria levels and the reduction in dental plaque accumulation after short-term (18–20) and long-term (12) usage of xylitol gum in many clinical studies. Previous clinical findings have indicated that the effective dose of xylitol is over 5–6 g per day with a chewing time of at least 5 min to significantly decrease the risk of caries (21). Additionally, a higher dose of xylitol does not have any additional improvement; instead, it may cause side effects such as diarrhea (22). However, few studies have investigated whether chewing xylitol decreases oral inflammation and changes the oral microbiota through third-generation sequencing technology.

Oral microbiota research has been conducted for diagnosis in the last decade. Comprehensive genomic profiling in dental caries using supragingival plaque has been investigated in children and elderly patients using next-generation sequencing (NGS) technology (23, 24). *Streptococcus* was found to be significantly increased in dental caries. However, the difficulty from previous studies was short-length read assembly with lower resolution despite the lower cost (25). The PacBio platform involves third-generation sequencing (PacBio SMRT; Pacific Biosciences of California, Inc., Menlo Park, CA, United States) for accurate analysis with the longest read lengths in microbial communities (26, 27). The error rate can be effectively reduced to 1% by single-molecule circular consensus sequencing (28).

Although several xylitol-related clinical experiments have been performed, there have not been many comprehensive investigations of the oral microbiota among young adults using a strict protocol (29, 30). In this study, we hypothesized that xylitol could influence cariogenic and periodontopathic bacterial growth in the oral cavity, which could later influence the oral microbiota. A randomized controlled trial (RCT) was designed to investigate microbiota changes using PacBio platform third-generation sequencing in young adults after chewing xylitol gum and those who did not chew gum for 2 weeks.

## Materials and Methods

### Participant Screening and Recruitment

For initial screening, each 2 (mL) of saliva was collected from 100 volunteers. The sample was first diluted 50-fold with phosphate-buffered saline (PBS) and further diluted 10-fold serial dilution for several times. Afterward, 50 μl of diluted saliva was plated on Mitis Salivarius-Bacitracin (MSB) agar and incubated for 3 days in an anaerobic chamber (37°C, 5% CO_2_ plus 95% N_2_) following bacterial colony counts. The volunteers were required to have at least 10^5^ CFU/mL *S. Mutant* colonization in the saliva, representing a high risk for dental caries (31).

After screening, we found 24 young adults (*N* = 24) participants were qualified for the study. All participants had more than 20 teeth, provided written informed consent, and were asked not to take any medication for 3 days before the experiment. Participants were excluded if they had a systemic disease or conditions (e.g., diabetes, cancer, cardiovascular disease), localized pain (e.g., localized abscess, localized infection), pregnant women, habitual consumers of xylitol-containing products, smoking, or alcohol. The decayed, missing, and filled teeth (DMFT) index was calculated, and full mouth scaling was offered 2 weeks prior to the baseline stage for clinical examination.

**FIGURE 1:**
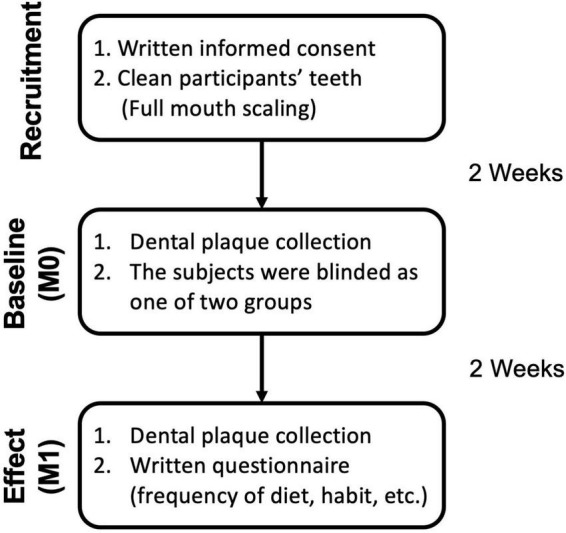
Sample collection flowchart and investigation schedule for subjects. After the initial washout period, participants were randomly assigned into one of two groups (Control with no-gum chewing, xylitol gum). Supragingival dental plaque was collected at baseline and 2 weeks after the treatment period for assessment. The questionnaire was shown in Supplementary Table 1.

Subsequently, baseline (M0) and effect (M1) dental plaque samples were collected for further examination. The flowchart is shown in Figure 1. The protocol was approved by the Taipei Medical University Joint Institutional Review Board (Approval No. N201910042) and followed the guidelines of the Strengthening the Reporting of Observational Studies in Epidemiology (STROBE) statement. All participants were randomly assigned into one of two groups after baseline measurement.

### Chewing Gum Selection

Xylitol gum (King Car Industrial Co., Ltd., Taipei, Taiwan) contained 0.62 g/pellet of xylitol, and the gum base included mint flavoring, soy lecithin, glycerin, titanium dioxide, and carnauba wax. The chewing gum was labeled with a letter on the package to differentiate between them.

### Study Protocol

Each participant was requested to allocate 1 month for this clinical study and use the same brand of toothpaste throughout the study period. In addition, the use of mouthwash and other xylitol-related products during this period was forbidden. After the washout period, subjects were randomly classified into one of two groups: a control group (no gum chewing) and xylitol gum chewing group. All subjects received a 2-week “washout period” during which no gum was chewed after full mouth scaling, followed by a 2-week treatment period. The subjects in the chewing gum group were asked to chew two pieces per time, five times every day, and at least 5 min for each chewing (30). The subjects in the control group were requested to brush twice a day, using the same brand of fluoride-containing toothpaste as the chewing gum group, but without chewing any gum during the whole period. The assessment of the effect on dental plaque was recorded after a 2-week trial.

### Sample Collection

The total period of this study was 4 weeks. For the baseline (M0) and effect (M1) examinations, subjects were asked to refrain from the following: brushing their teeth for at least 48 h, eating at least 6 h before the examination, and drinking at least 2 h before the examination. Gracey curettes were used to collect the supragingival plaque from all teeth of each subject without activating it on the tooth’s buccal and lingual/palatal surfaces. Curettes were sterilized before use, and the samples were stored in a sterile Eppendorf tube for further analysis. After the sampling was completed, the sample was labeled according to the grouping. Supragingival plaque samples were analyzed for weight, pH value, and 16S rDNA full-length sequencing.

### Experimental Design

This study mainly followed the “Estimate the Health Food Effect for Dental Health” guidelines, which were released by the Ministry of Health and Welfare (MOHW) (31). Changes in dental plaque accumulation, pH value, and 16S full-length rDNA sequencing were assessed for the effects on subjects’ dental health. At baseline (M0) and 2 weeks after baseline (M1), the dental plaque from all teeth of each subject was collected, weighed for plaque accumulation, and measured for pH value. The dental plaque weight was evaluated using a digital electronic microbalance, and the pH value was determined using a micro pH electrode (Orion Model 9863BN, Thermo Scientific, Chelmsford, MA, United States) to estimate the caries susceptibility.

For 16S rDNA full-length amplicon sequencing with the PacBio SMRT sequencing platform, plaque was extracted and prepared according to the manufacturer’s protocol of the GenElute™ Bacterial Genomic DNA Kit (Sigma-Aldrich, St. Louis, MO, United States). The final DNA solution from this sample was collected with an elution solution (10 mM Tris–HCl, 0.5 mM EDTA, pH 9.0) for further sequencing. The full-length 16S rDNA sequence was amplified using the bacterial-specific universal PCR primers 27F and 1492R according to the manufacturer’s instructions. The raw reads were processed using pbccs (v.3.4.0) in SMRT Link Analysis v6.0.0, which generated consensus sequences with low error rates. Sequences for each sample were processed using the software package QIIME version 1.9.0 (32). To avoid possible sequencing errors, operational taxonomic unit (OTU) picking was performed using mothur v.1.39.5 with 97% identity (33). Chimeric sequences were identified using UCHIME v4.2 with the Gold database and made OTU table (34). The microbial diversity analyses of our samples were based on the Human Oral Microbiome Database (HOMD) v15.2 using QIIME.

We chose IBM SPSS, version 19 (IBM Co., Armonk, NY, United States) for further statistical analysis and compared the difference between effect and baseline assessments in each group. The effect of the gum was assessed using the Student’s *t*-test or Wilcoxon test. The microbial community analyses from the OTUs were performed using the Kruskal–Wallis test and Wilcoxon test, when appropriate.

## Results

The background and dental information from all participants are shown in Table 1. There were 13 subjects in the xylitol gum group and 11 subjects in the control group. The average participants’ age range was 23–25 years, and the DMFT index ranged from 9 to 10. Age and DMFT index were not significantly different between the two groups because the present study is a randomized clinical trial. The demographics and specific information of participants were listed in Supplementary Table 2.

**TABLE 1:** Characteristics of participants from the control and xylitol gum groups.

Basic profile	Control	Xylitol gum
Sample size, *N*	11	13
Male: Female	6:5	6:7
Age, years	24.91 ± 3.91	23.31 ± 3.12
Decayed, Missing, and Filled Teeth (DMFT) index	10 (8–13)	9 (6–13)
Number of decayed teeth (DT value)	2 (1–3)	1 (1–4)
Number of missing teeth (DM value)	3 (2–4)	4 (1–4)
Number of filled teeth (DF value)	4 (2–9)	3 (1–5)

*There was no statistically significant difference between the two groups.*

The average dental plaque weight, pH value, unique tags, Good’s coverage, and alpha diversity are shown in Table 2. At least 10,000 effective unique tags were classified into OTUs, and 99.0% of Good’s coverage remained in all groups (Supplementary Table 3). Thus, the sequencing depth was sufficient to recover the oral bacterial communities. Interestingly, the present data showed that chewing xylitol gum for 2 weeks (at M1) resulted in a 19% significant reduction in dental plaque weight (*p* < 0.05), while controls had an increase of 7%. In addition, the Shannon diversity index of the microbiota in the xylitol gum group significantly decreased after the trial due to the reduction in pathological microorganisms (*p* < 0.05), but no significant difference was observed in the effects in the control group. The effect on plaque samples in the xylitol gum group was reduced weight. A smaller amount of dental plaque was presented with a small taxonomically diversity than the control group. However, the average pH value in both groups remained within the healthy range with no significant change before and after the study period.

**TABLE 2:** Average dental plaque weight, pH value from the two groups at M0 and M1, respectively.

Variable	Control		Xylitol gum	
	M0	M1	M0	M1
Average dental plaque weight, mg	32.00	34.30	34.80	26.40*
	(19.15**–**66.60)	(22.10**–**49.50)	(30.50**–**57.60)	(16.17**–**37.80)
Average pH value	6.53	6.58	6.55	6.58
	(6.35–6.79)	(6.49–6.80)	(6.28–6.79)	(6.30–6.67)

*p < 0.05 by Wilcoxon’s test; The average dental plaque weight was significantly decreased following chewing xylitol gum treatment.

**FIGURE 2:**
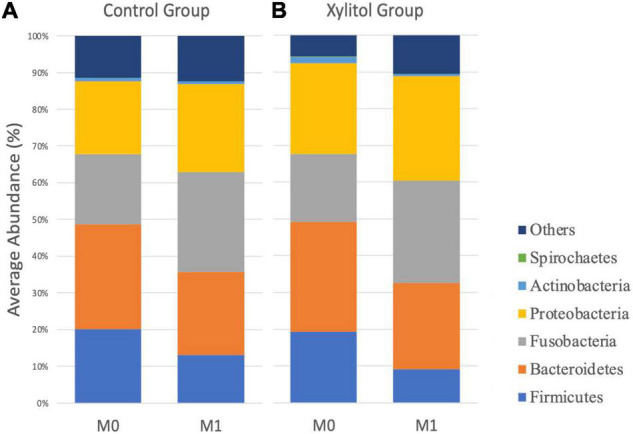
The average profile of OTUs of oral dental plaque at the phylum level at M0 and M1 from the **(A)** control group and **(B)** xylitol gum group.

Figure 2A shows the histogram of the relative abundance of oral bacterial phyla at M0 and M1 for the two groups. In the control group, the fluoride-containing toothpaste effect decreased the average abundance of *Firmicutes and Bacteroidetes* from 20.14 and 28.49% at baseline to 13.02 and 22.62%, respectively. The average abundance of *Proteobacteria* slightly increased from 19.80 to 23.89%, and that of *Fusobacteria* significantly increased from 19.12% at baseline to 27.28% after 2 weeks (*p* < 0.05). The results from the control group indicated that regular tooth brushing with fluoride-containing toothpaste and improved oral hygiene behaviors would have a slightly beneficial effect on dental health.

**FIGURE 3:**
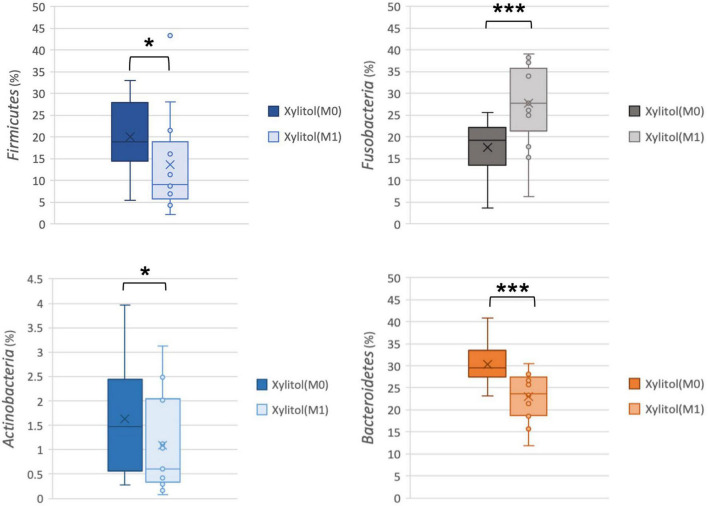
Comparison of relative abundance at the phylum level from the xylitol gum group at M0 and M1; *p < 0.05, and ***p < 0.001 respectively, using Wilcoxon’s test.

In Figures 2B, 3, the xylitol gum group shows a decrease in oral *Firmicutes*, and *Bacteroidetes* from 19.28 and 29.90% to 9.02 (*p* < 0.05) and 23.58% (*p* < 0.001), respectively. Notably, the average abundance of *Actinobacteria* was significantly decreased by 1.66% after 2 weeks of chewing xylitol gum (*p* < 0.05). The average abundance of *Fusobacteria* was increased by 6% after 2 weeks in the xylitol gum group (*p* < 0.001). The xylitol group had a significant increase (*p* < 0.001) in the relative abundance of *Fusobacteria* compared to that in the control group (*p* < 0.05).

**FIGURE 4:**
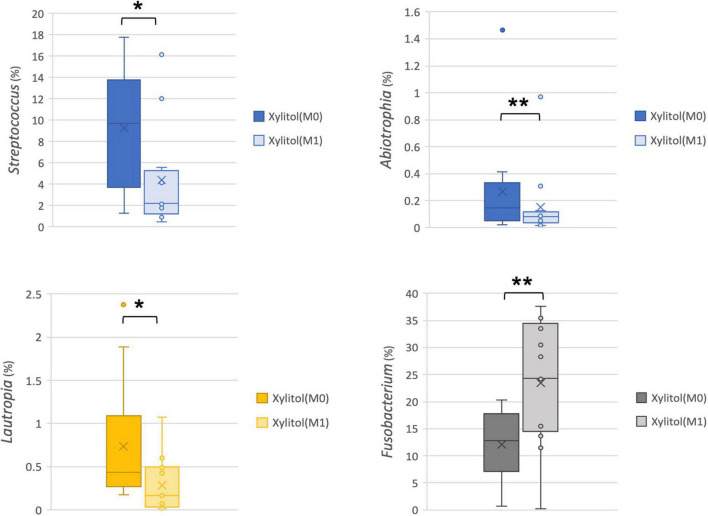
Comparison of the relative abundance of cariogenic bacteria at the family or genus level from the xylitol gum group at M0 and M1; *p < 0.05, **p < 0.01, respectively, using Wilcoxon’s test.

**FIGURE 5:**
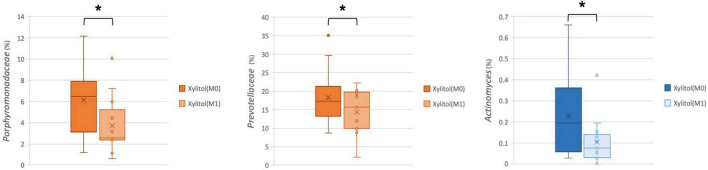
Comparison of the relative abundance of periodontopathic bacteria at the family or genus level in the xylitol gum group at M0 and M1; *p < 0.05, respectively, using Wilcoxon’s test.

In addition, the cariogenic and periodontopathic bacteria at the family or genus level from the xylitol gum group are shown in Figures 4, 5. This result indicated a significantly decreased tendency of *Streptococcus* (*p* < 0.05), *Lautropia* (*p* < 0.01), and *Abiotrophia* (*p* < 0.05) (nutritionally variant streptococci), all of which are related to dental caries. Additionally, a lower abundance (*p* < 0.05) of *Prevotellaceae*, *Porphyromonas*, and *Actinomyces* after 2 weeks of chewing xylitol gum was observed, these bacteria are associated with periodontal disease. In more detail, the result of the major species-level bacterial community from control and xylitol groups were listed in Table 3. Taxa detected periodontopathic bacteria of *Actinomyces* spp., *Actinomyces gerencseriae*, *Actinomyces meyeri*, *Actinomyces odontolyticus*, *Porphyromonas* sp., *Alloprevotella sp._HMT_914*, *Porphyromonas* spp., *Porphyromonas sp._HMT_278*, *Fusobacterium periodonticum* were significantly decreased in xylitol gum group than control group. Similarly, cariogenic microorganisms of *Abiotrophia defective*, *Streptococcus anginosu*, *Streptococcus gordonii*, *Streptococcus intermedius*, *Streptococcus oralis*, *Streptococcus salivarius*, *Lautropia mirabilis* were also decreased in xylitol gum group. These results indicated that only chewing xylitol gum exerts a suppressing effect on both cariogenic and periodontopathic microorganisms in the oral cavity after 2 weeks of use.

**TABLE 3:** Detection selected abundance of *Abiotrophia*, *Actinomyces*, *Porphyromonas*, *Alloprevotella*, *Porphyromonas*, *Streptococcus*, *Fusobacterium*, and *Leptotrichia* species carried by control and xylitol groups.

Species level	Control group				Xylitol group			
	Significance	Trend	Relative median abundance (%)		Significance	Trend	Relative median abundance (%)	
			M0	M1			M0	M1
*Abiotrophia defective* (*nutritionally variant Streptococci*)			0.336	0.258	**	↓	0.141	0.09
*Actinomyces unclassified* (*Actinomyces* spp.)			0.018	0.016	*	↓	0.026	0.01
*Actinomyces gerencseriae*			0.032	0.009	*	↓	0.01	0
*Actinomyces meyeri*			0.001	0.001	**	↓	0.02	0.003
Actinomyces odontolyticus			0.003	0.001	**	↓	0.008	0.005
*Actinomyces* sp._HMT_448			0.009	0.005	*	↓	0.026	0
*Porphyromonas unclassified* (*Porphyromonas* spp.)			0.358	0.279	***	↓	0.929	0.412
*Porphyromonas catoniae*			0.528	0.314			0.466	0.390
Porphyromonas sp._HMT_278					**	↓	0.041	0.018
Porphyromonas sp._HMT_284			0.007	0.006			0.008	0.004
*Alloprevotella* sp._HMT_914			0.123	0.107	**	↓	0.344	0.083
*Alloprevotella rava*			0.081	0.087			0.065	0.099
*Prevotella unclassified* (*Prevotella* spp.)			4.239	2.747			2.330	2.920
*Prevotella denticola*			0.081	0.146			0.127	0.119
*Prevotella histicola*			0.014	0.009			0.003	0
*Prevotella intermedia*			0.006	0.0			0.043	0.004
*Prevotella melaninogenica*			0.517	0.6			0.225	0.273
*Prevotella nigrescens*			0.247	0.263			0.172	0.157
*Prevotella oulorum*			0.055	0.072			0.326	0.149
*Prevotella pallens*			0.048	0.035			0.011	0.016
*Prevotella veroralis*			0.040	0.047			0.344	0.212
*Streptococcus unclassified* (*Streptococcus* spp.)			4.335	1.642			3.270	1.621
*Streptococcus anginosus*			0	0			0.031	0.013
*Streptococcus cristatus_clade_578*			0.030	0.013			0.051	0.029
*Streptococcus gordonii*			0.160	0.114	*	↓	0.197	0.039
*Streptococcus intermedius*			0	0			0.017	0.008
*Streptococcus oralis_subsp._dentisani_clade_058*			2.338	1.906			1.744	0.858
*Streptococcus oralis_subsp._dentisani_clade_398*			0	0			0.003	0.003
*Streptococcus oralis_subsp._tigurinus_clade_071*			0.078	0.145			0.177	0.172
*Streptococcus salivarius*			0.024	0			0.003	0
*Streptococcus sanguinis*			0.041	0.032			0.038	0.030
*Fusobacterium unclassified* (*Fusobacterium* spp.)			9.807	8.878	*	↑	8.541	10.199
*Fusobacterium naviforme*			0	0.001	*	↑	0	0.015
*Fusobacterium nucleatum_subsp._animalis*	*	↑	0.492	1.391	*	↑	1.774	3.697
*Fusobacterium nucleatum_subsp._polymorphum*			0.120	0.072			0.077	0.099
*Fusobacterium nucleatum_subsp._vincentii*	**	↑	0.359	1.500	**	↑	1.281	2.083
*Fusobacterium periodonticum*			0.958	0.598	*	↓	0.324	0.097
*Fusobacterium* p._HMT_203	*	↑	0.065	0.118			0.081	0.116
*Lautropia mirabilis*			0.840	0.330	***	↓	0.446	0

*p < 0.05, **p < 0.01, and ***p < 0.001 by Wilcoxon’s test.

**FIGURE 6:**
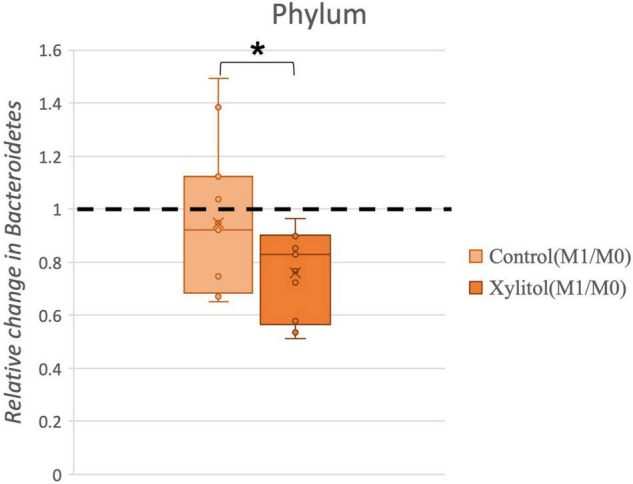
Comparison of the relative change in *Bacteroidetes* in the control and xylitol groups. The value of abundance of *Bacteroidetes* for M1 was divided by M0. Error bars indicate the interquartile values, and the dashed line represents the baseline (M0/M0 = 1); “*” indicates *p* < 0.05 when compared with the baseline using the two-tailed Wilcoxon test.

To account for individual physiological differences, the relative change index (%, effectiveness/baseline) from oral bacteria was calculated for the xylitol gum and control groups. Figure 6 shows a significant relative change (*P* < 0.05) for these two groups, which indicates that chewing xylitol gum significantly suppressed the growth of *Bacteroidetes* compared to that of the control group.

**FIGURE 7:**
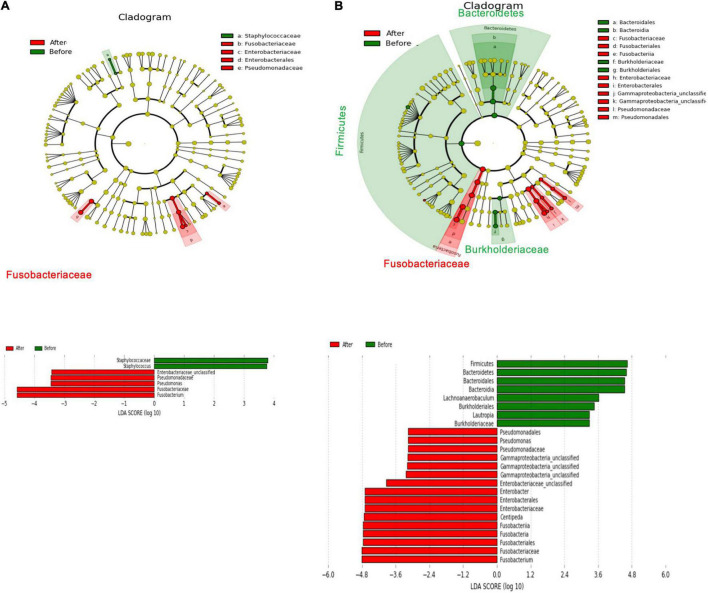
The main abundant taxa that differed before treatment (M0, baseline) and after the 2-week follow-up (M1) in the taxonomic cladogram based on Linear discriminant analysis effect size (LEfSe) analysis and its LDA score from the **(A)** control group **(B)** xylitol gum group.

**FIGURE 8:**
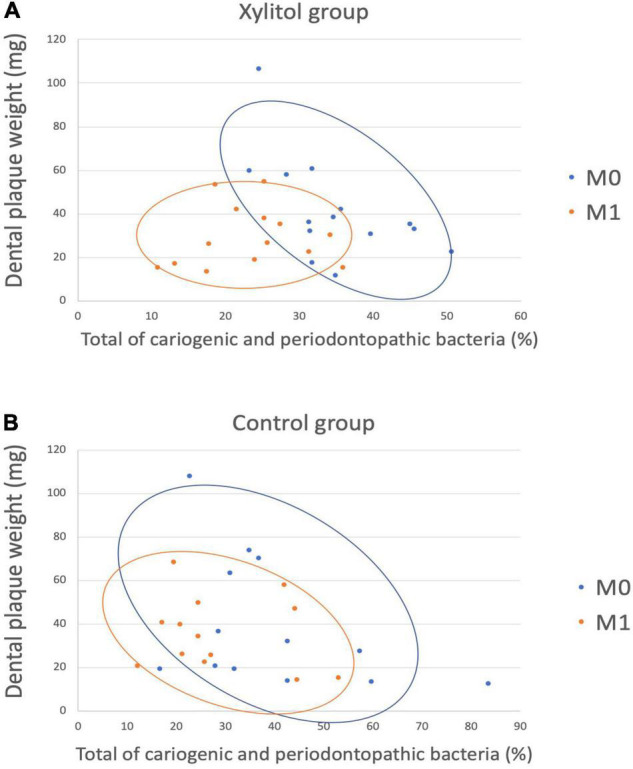
A scatter plot showing the distribution between dental plaque accumulation and a total of cariogenic and periodontopathic bacteria among the **(A)** Xylitol and **(B)** Control group, showing the confidence ellipses (51). Total of cariogenic and periodontopathic bacteria and dental plaque accumulation are plotted on the horizontal and vertical axes, respectively. Each blue dots represent baseline (M0), and the orange dots represent M1.

In Figure 7A, a linear discriminant analysis effect size-based taxonomic cladogram depicts successive circles that represent a phylogenetic level and a linear discriminant analysis (LDA) bar graph. The brightness of each dot is proportional to its effect size, and each LDA bar length was represented by a log_10_ transformed LDA. The taxa that met a significant LDA threshold value of >| ± 3| are shown. Only the species in the family *Fusobacteriaceae* were enriched in the control group (Figure 7A). The species in the phylum *Firmicutes*, phylum *Bacteroidetes*, phylum *Fusobacteria*, class *Burkholderiaceae*, class *Fusobacteriia*, and genus *Lautropia* were enriched in the xylitol gum group (Figure 7B). Similarly, Figure 8 shows a region of oral health distribution by confidence ellipses plotting the total cariogenic, periodontopathic bacteria and dental plaque accumulation. After 2 weeks of chewing xylitol gum (Figure 8A), most of the dots are found in the lower left area representing the effectiveness of xylitol inducing less dental plaque, and less total bacteria count. On the other hand, a slight change after 2 weeks in control group (Figure 8B).

## Discussion

Over the years, humans have consumed various products for pleasure and health, including chewing gum (35). For decades, dentists discouraged the use of chewing gum due to sucrose additives. However, evidence showed that chewing gum would promote enamel remineralization by stimulating saliva flow and increasing plaque pH (36, 37). Chewing gum can be easily found with non-sugar sweeteners such as xylitol. The present study investigated the effect of xylitol chewing gum on dental plaque accumulation and its microbiota composition using the PacBio sequencing platform in 34 young adults. All subjects recruited in the study had a similar taxonomic composition when comparing the number of caries, missing teeth, and the DMFT index (Tables 1, 2). Notably, chewing xylitol gum was significantly reduced the weight of dental plaque and the abundance of pathogenic bacteria compared to the control group (Table 2 and Supplementary Table 2). Our results revealed a statistically significant difference in the plaque weight in the xylitol gum group (19% reduction) compared to the control group (*p* < 0.05). Moreover, subjects in the control group had 7% higher dental plaque weight than their baseline (Table 2). The increased dental plaque weight indicated a higher number of bacteria adhere to the tooth surface and the posterior biofilm formation that led to dental caries and periodontal disease.

The caries process comprises acidogenic and aciduric bacteria that are responsible for lowering the pH value and subsequent destruction of the hydroxyapatite matrix in enamel and dentine (38). In the present study, chewing xylitol gum decreased the abundance of *Streptococcus*, *Lautropia*, and *Abiotrophia* (nutritionally variant *Streptococci*) (Figure 4). These cariogenic microorganisms are associated with the cause or progression of the dental lesion (39). In the present study, *Streptococcus* levels had a statistically significant decrease (*p* < 0.05) between baseline and 2 weeks after the xylitol gum trial, which could prevent caries initiation or progression. This seemed to be related to the reduction of sucrose, increase in salivary flow and tooth surface self-cleaning during mastication. Xylitol’s benefits can lead to a significantly lower total bacterial percentage can be seen as early as a 2-days experimental duration according to a study published in 2018 (40). In their study, researchers have also found an effect of xylitol-containing chewing gum on the oral microbiota, but no significant difference was observed in the overall composition of the salivary microbiota between the baseline and follow-up samples of the two groups, including participants aged 19–52 years with a DMFT index from 0 to 24. In addition, the pH value of the dental plaque from our results was still considered healthy after 2 weeks, which showed 6.50 and 6.21 pH levels, respectively.

To the best of our knowledge, not only caries (41) but also patients with periodontitis (42, 43) have higher abundance levels of *Bacteroidetes* than healthy individuals. In the present study, only the xylitol gum group reduced the abundance of *Actinobacteria* (*Actinomyces* spp., *A. gerencseriae*, *A. meyeri*, *A. odontolyticus*), *Prevotellaceae*, and *Porphyromonas* (*Porphyromonas* spp., *P. sp._HMT_278*), but this reduction was not observed in the control group (Figure 3). The same tendency was previously described by He et al. (41) in their taxonomic and functional analyses of the supragingival microbiome from caries-affected and caries-free hosts. Their study also found that the family *Prevotellaceae* had a higher relative abundance in the supragingival dental plaque of caries patients, although the difference was not statistically significant (41). Importantly, the average abundance of *Prevotellaceae* (*P. intermedia*, *P. nigrescens*, *P. oulorum*, *P. veroralis*) in our study decreased at the family and species levels after subjects chewed xylitol for 2 weeks (Figure 5 and Table 3). This evidence suggests that the effectiveness of xylitol can reduce dental plaque and relieve gingival inflammation. Xylitol has only been reported to inhibit the gene expression of the inflammatory cytokines TNF-α and IL-1β in *in vitro* studies (44). Therefore, our result of xylitol’s clinical effect in microbiota suggests that chewing xylitol gum may have not only a potentially positive anti-inflammatory effect in the oral cavity but also bacteria inhibition growth, which was not previously investigated in human studies through high-throughput 16S rDNA full-length amplicon sequencing (45, 46). These data provide valuable insights into preventing oral infections such as caries and periodontal diseases by chewing xylitol gum.

The PacBio sequencing platform was used to estimate the mean percentage of supragingival plaque microbiota from each group. Our study found that only had *Fusobacterium* significantly increased in the control group. The average abundance of *Firmicutes*, *Bacteroides*, and *Actinobacteria* significantly decreased, while that of *Fusobacterium* significantly increased in the xylitol gum group. Similar studies have also suggested that *Fusobacterium* and *Pasteurellaceae* constituted a higher proportion of dental plaque in healthy adults (47, 48). According to our present findings, only xylitol gum had a beneficial effect on dental health. The xylitol effectiveness was evaluated to reveal its clinical advantages in the preventive oral health approach. Whereas the control group did not experience any significantly higher tendency of caries and periodontal disease. These findings can be related to the longer study period, and our study participants were recruited between ages 20–30 years with high DMFT levels. Referencing the different findings among previous studies (49) and our study, the level of the DMFT index might be related to the detectability of the benefits from chewing xylitol-containing gum. The higher the DMFT level, the more significant the clinical effect of xylitol in decreasing cariogenic and periodontopathic bacteria in dental plaque. In subjects with good oral hygiene, xylitol gum will have the same benefits for reducing the risk of caries as conventional chewing gum, such as medical reasons including halitosis and xerostomia reduction, aid to digestion, and psychological help like nervous tension relief (35). Last, the *Firmicutes*/*Bacteroidetes* (F/B ratio) value from all groups was less than one, which indicates no intense dysbiosis or inflammatory reaction during the study (50).

This study employed the full-length of a 16S rDNA PacBio SMRT sequence to evaluate the effectiveness of chewing gum for short-term use. This result indicated that xylitol gum had an excellent anti-cariogenic and anti-periodontopathic clinical effect based on oral microbiota modulation. However, there are still some limitations that could be improved in the future. First, a longer-term treatment over 1 month of observation was not performed in this study. Second, this method preserves the significance of differences in abundance from microbial communities. However, the increased sensitivity for specific bacteria should still be used in the traditional approach (i.e., laboratory culture-based colonization of selective media). Finally, adverse effects on the TMJ, jaw muscle pain, and even though none of the participants had diarrhea, it may occur with excessive chewing of xylitol gum (22).

## Conclusion

In conclusion, our study showed statistically significant differences in dental plaque accumulation and associated microbial communities after chewing xylitol gum, as determined by the PacBio SMRT sequencing platform. The results showed that dental plaque accumulation decreased due to bacterial adherence, the relative abundance of cariogenic bacteria such as *Streptococcus*, *Lautropia*, and *Abiotrophia* and that of periodontopathic bacteria such as *Porphyromonas*, *Actinomyces*, and *Prevotellaceae* bacteria were significantly decreased by daily chewing of xylitol gum (intake of 10 pellets for a total of 6.2 g xylitol/day) for 2 weeks. Xylitol gum appeared to reduce the likelihood of caries and the development of periodontal disease by limiting bacterial growth. Based on our findings, no similar studies have employed third-generation sequencing technology, which could be used in future studies for more precise results.

## Data Availability Statement

The 16S rDNA data sets generated in this study were deposited in the EBI European Nucleotide Archive database (European Bioinformatics Institute, Cambridge, United Kingdom) under the accession number: PRJEB51987.

## Ethics Statement

The studies involving human participants were reviewed and approved by Taipei Medical University Joint Institutional Review Board (Approval No. N201910042). The patients/participants provided their written informed consent to participate in this study.

## Author Contributions

N-CT and W-JC conceived and designed the experiments. Y-FW, ES, I-WC, J-NS, Y-CC, and SW performed the experiments. Y-FW, Y-CC, and SW analyzed the data. Y-FW, ES, I-WC, J-NS, SW, and W-JC wrote the manuscript. Y-SS, N-CT, and W-JC developed this study. All authors contributed to the article and approved the submitted version.

## Conflict of Interest

The authors declare that the research was conducted in the absence of any commercial or financial relationships that could be construed as a potential conflict of interest.

## Publisher’s Note

All claims expressed in this article are solely those of the authors and do not necessarily represent those of their affiliated organizations, or those of the publisher, the editors and the reviewers. Any product that may be evaluated in this article, or claim that may be made by its manufacturer, is not guaranteed or endorsed by the publisher.

## References

[1] Petersen PE. The world oral health report 2003: continuous improvement of oral health in the 21st century–the approach of the who global oral health programme. *Commun Dent Oral Epidemiol.* (2003) 31:3–24. 10.1046/j..2003.com122.x 15015736

[2] Wong A Subar PE Young DA. Dental caries: an update on dental trends and therapy. *Adv Pediatr.* (2017) 64:307–30. 10.1016/j.yapd.2017.03.011 28688595

[3] Yadav K Prakash S. Dental caries: a microbiological approach. *J Clin Infect Dis Pract.* (2017) 2:1–15.

[4] Tanzer JM Livingston J Thompson AM. The microbiology of primary dental caries in humans. *J Dent Educ.* (2001) 65:1028–37. 10.1002/j.0022-0337.2001.65.10.tb03446.x 11699974

[5] Cronin M Gordon J Reardon R Balbo F. Three clinical trials comparing xylitol-and sorbitol-containing chewing gums for their effect on supragingival plaque accumulation. *J Clin Dent.* (1994) 5:106–9. 8602901

[6] Keijser B Zaura E Huse S Van der Vossen J Schuren F Montijn R Pyrosequencing analysis of the oral microflora of healthy adults. *J Dent Res.* (2008) 87:1016–20. 10.1177/154405910808701104 18946007

[7] Surdacka A Stopa J. The effect of xylitol toothpaste on the oral cavity environment. *J Prev Med.* (2005) 13:98–107.

[8] Tanzer JM Clive J. Quantitative considerations in microbiological evaluations for caries: risks for type ii errors resulting from use of MSB agar. *Oral Microbiol Immunol.* (1986) 1:28–30. 10.1111/j.1399-302x.1986.tb00313.x

[9] Hajiahmadi M Yegdaneh A Homayoni A Parishani H Moshkelgosha H Salari-Moghaddam R. Comparative evaluation of efficacy of “green tea” and “green tea with xylitol” mouthwashes on the salivary *Streptococcus Mutans* and *Lactobacillus* colony count in children: a randomized clinical trial. *J Contemp Dent Pract.* (2019) 20:1190–4. 10.5005/jp-journals-10024-2652 31883255

[10] Ur-Rehman S Mushtaq Z Zahoor T Jamil A Murtaza MA. Xylitol: a review on bioproduction, application, health benefits, and related safety issues. *Crit Rev Food Sci Nutr.* (2015) 55:1514–28. 10.1080/10408398.2012.702288 24915309

[11] Fischer E Stahel R. *Zur Kenntnis Der Xylose. Untersuchungen Über Kohlenhydrate Und Fermente (1884–1908).* Berlin: Springer (1909). p. 389–99.

[12] Nayak PA Nayak UA Khandelwal V. The effect of xylitol on dental caries and oral flora. *Clin Cosmet Investig Dent.* (2014) 6:89. 10.2147/CCIDE.S55761 25422590 PMC4232036

[13] Imfeld T. Chewing gum—facts and fiction: a review of gum-chewing and oral health. *Crit Rev Oral Biol Med.* (1999) 10:405–19. 10.1177/10454411990100030901 10759416

[14] Söderling E Mäkinen K Chen CY Pape HR Jr Loesche W Mäkinen PL. Effect of sorbitol, xylitol, and xylitol/sorbitol chewing gums on dental plaque. *Caries Res.* (1989) 23:378–84. 10.1159/000261212 2766327

[15] Sato Y Yamamoto Y Kizaki H. Xylitol-induced elevated expression of the GBPC gene in a population of *Streptococcus mutans* cells. *Eur J Oral Sci.* (2000) 108:538–45. 10.1034/j.1600-0722.2000.00928.x 11153929

[16] Ylikahri R. Metabolic and nutritional aspects of xylitol. *Adv Food Res.* (1979) 25:159–80. 10.1016/s0065-2628(08)60237-2 391001

[17] Authority EFS. Xylitol chewing gum/pastilles and reduction of the risk of tooth decay-scientific substantiation of a health claim related to xylitol chewing gum/pastilles and reduction the risk of tooth decay pursuant to article 14 of regulation (Ec) No 1924/2006-scientific opinion of the panel on dietetic products, nutrition and allergies. *EFSA J.* (2008) 6:852. 10.2903/j.efsa.2008.852

[18] Seki M Karakama F Kawato T Tanaka H Saeki Y Yamashita Y. Effect of Xylitol Gum on the Level of Oral mutans Streptococci of preschoolers: block-randomised trial. *Int Dent J.* (2011) 61:274–80. 10.1111/j.1875-595X.2011.00073.x 21995376 PMC9374824

[19] Shinga-Ishihara C Nakai Y Milgrom P Söderling E Tolvanen M Murakami K. Xylitol carryover effects on salivary mutans Streptococci after 13 months of chewing xylitol gum. *Caries Res.* (2012) 46:519–22. 10.1159/000341221 22890503

[20] Loesche WJ Grossman NS Earnest R Corpron R. The effect of chewing xylitol gum on the plaque and saliva levels of *Streptococcus Mutans*. *J Am Dent Assoc.* (1984) 108:587–92. 10.14219/jada.archive.1984.0390 6427315

[21] Campus G Cagetti MG Sale S Petruzzi M Solinas G Strohmenger L Six months of high-dose xylitol in high-risk caries subjects—a 2-year randomised, clinical trial. *Clin Oral Investig.* (2013) 17:785–91. 10.1007/s00784-012-0774-5 22791282 PMC3607712

[22] Vernacchio L Vezina RM Mitchell AA. Tolerability of oral xylitol solution in young children: implications for otitis media prophylaxis. *Int J Pediatr Otorhinolaryngol.* (2007) 71:89–94. 10.1016/j.ijporl.2006.09.008 17097152 PMC1780176

[23] Jiang W Ling Z Lin X Chen Y Zhang J Yu J Pyrosequencing analysis of oral microbiota shifting in various caries states in childhood. *Microb Ecol.* (2014) 67:962–9. 10.1007/s00248-014-0372-y 24504329

[24] Jiang Q Liu J Chen L Gan N Yang D. The oral microbiome in the elderly with dental caries and health. *Front Cell Infect Microbiol.* (2019) 8:442. 10.3389/fcimb.2018.00442 30662876 PMC6328972

[25] Orkunoglu-Suer F Harralson AF Frankfurter D Gindoff P O’Brien TJ. Targeted Single molecule sequencing methodology for ovarian hyperstimulation syndrome. *BMC Genomics.* (2015) 16:264. 10.1186/s12864-015-1451-2 25888426 PMC4397691

[26] Ardui S Ameur A Vermeesch JR Hestand MS. Single molecule real-time (Smrt) sequencing comes of age: applications and utilities for medical diagnostics. *Nucleic Acids Res.* (2018) 46:2159–68. 10.1093/nar/gky066 29401301 PMC5861413

[27] Zhang W Sun Z Menghe B Zhang H. Single molecule, real-time sequencing technology revealed species-and strain-specific methylation patterns of 2 *Lactobacillus* strains. *J Dairy Sci.* (2015) 98:3020–4. 10.3168/jds.2014-9272 25747834

[28] Wagner J Coupland P Browne HP Lawley TD Francis SC Parkhill J. Evaluation of PacBio sequencing for full-length bacterial 16s rRNA gene classification. *BMC Microbiol.* (2016) 16:274. 10.1186/s12866-016-0891-4 27842515 PMC5109829

[29] Söderling E Hirvonen A Karjalainen S Fontana M Catt D Seppä L. The effect of xylitol on the composition of the oral flora: a pilot study. *Eur J Dent.* (2011) 5:24. 21311610 PMC3037192

[30] Kandelman D Gagnon G. Clinical results after 12 months from a study of the incidence and progression of dental caries in relation to consumption of chewing-gum containing xylitol in school preventive programs. *J Dent Res.* (1987) 66:1407–11. 10.1177/00220345870660082501 3476611

[31] Wang YB Chuang CY Liao JF. Effects of xylitol in chewing gum on dental plaque and *Streptococcus mutans*. *J Food Anal.* (2006) 14:84–8.

[32] Caporaso JG Kuczynski J Stombaugh J Bittinger K Bushman FD Costello EK Qiime allows analysis of high-throughput community sequencing data. *Nat Methods.* (2010) 7:335–6. 10.1038/nmeth.f.303 20383131 PMC3156573

[33] Blaxter M Mann J Chapman T Thomas F Whitton C Floyd R Defining operational taxonomic units using DNA barcode data. *Philos Trans R Soc B Biol Sci.* (2005) 360:1935–43. 10.1098/rstb.2005.1725 16214751 PMC1609233

[34] Haas BJ Gevers D Earl AM Feldgarden M Ward DV Giannoukos G Chimeric 16s rRNA sequence formation and detection in sanger and 454-pyrosequenced PCR amplicons. *Genome Res.* (2011) 21:494–504. 10.1101/gr.112730.110 21212162 PMC3044863

[35] Hartel RW Joachim H Hofberger R. *Confectionery Science and Technology.* Cham: Springer (2018). p. 393–420.

[36] Twetman S. Consistent evidence to support the use of xylitol-and sorbitol-containing chewing gum to prevent dental caries. *Evid Based Dent.* (2009) 10:10–1. 10.1038/sj.ebd.6400626 19322219

[37] Nakagawa K Watanabe S Pai C Minami M Suzuki A Takamori K. The concentration of gum component in saliva before and after swallowing during prolonged gum chewing. *Pediatr Dent J.* (2007) 17:79–83. 10.1016/s0917-2394(07)70099-x

[38] Kianoush N Nguyen KAT Browne GV Simonian M Hunter N. pH gradient and distribution of Streptococci, Lactobacilli, Prevotellae, and Fusobacteria in carious dentine. *Clin Oral Investig.* (2014) 18:659–69. 10.1007/s00784-013-1009-0 23771212 PMC3883984

[39] Loesche WJ. Microbiology of dental decay and periodontal disease. 4th ed. In: Baron S editor. *Medical Microbiology.* (Galveston, TX: University of Texas Medical Branch at Galveston) (1996). 21413316

[40] Takeuchi K Asakawa M Hashiba T Takeshita T Saeki Y Yamashita Y. Effects of xylitol-containing chewing gum on the oral microbiota. *J Oral Sci.* (2018) 60:588–94. 10.2334/josnusd.17-0446 30429438

[41] He J Tu Q Ge Y Qin Y Cui B Hu X Taxonomic and functional analyses of the supragingival microbiome from caries-affected and caries-free hosts. *Microbial Ecol.* (2018) 75:543–54. 10.1007/s00248-017-1056-1 28932895

[42] Chen WP Chang SH Tang CY Liou ML Tsai SJJ Lin YL. Composition analysis and feature selection of the oral microbiota associated with periodontal disease. *Biomed Res Int.* (2018) 2018:3130607. 10.1155/2018/3130607 30581850 PMC6276491

[43] Rodrigues MX Bicalho RC Fiani N Lima SF Peralta S. The subgingival microbial community of feline periodontitis and gingivostomatitis: characterization and comparison between diseased and healthy cats. *Sci Rep.* (2019) 9:1–10. 10.1038/s41598-019-48852-4 31451747 PMC6710259

[44] Han SJ Jeong SY Nam YJ Yang KH Lim HS Chung J. Xylitol inhibits inflammatory cytokine expression induced by lipopolysaccharide from *Porphyromonas gingivalis*. *Clin Diagn Lab Immunol.* (2005) 12:1285–91. 10.1128/CDLI.12.11.1285-1291.2005 16275942 PMC1287760

[45] Yin SY Kim HJ Kim HJ. Protective effect of dietary xylitol on influenza a virus infection. *PLoS One.* (2014) 9:e84633. 10.1371/journal.pone.0084633 24392148 PMC3879333

[46] Páyer E Szabó-Papp J Ambrus L Szöllõsi AG Andrási M Dikstein S Beyond the physico-chemical barrier: glycerol and xylitol markedly yet differentially alter gene expression profiles and modify signalling pathways in human epidermal keratinocytes. *Exp Dermatol.* (2018) 27:280–4. 10.1111/exd.13493 29520873

[47] Cockburn AF Dehlin JM Ngan T Crout R Boskovic G Denvir J High throughput DNA sequencing to detect differences in the subgingival plaque microbiome in elderly subjects with and without dementia. *Investig Genet.* (2012) 3:1–12. 10.1186/2041-2223-3-19 22998923 PMC3488532

[48] Borkent D Reardon R McLachlan G Glendinning L Dixon P. A microbiome analysis of equine peripheral dental caries using next generation sequencing. *Equine Vet J.* (2020) 52:67–75. 10.1111/evj.13126 31006119

[49] Rafeek R Carrington CV Gomez A Harkins D Torralba M Kuelbs C Xylitol and sorbitol effects on the microbiome of saliva and plaque. *J Oral Microbiol.* (2019) 11:1536181. 10.1080/20002297.2018.1536181 30598728 PMC6225370

[50] Wu YF Lee WF Salamanca E Yao WL Su JN Wang SY Oral microbiota changes in elderly patients, an indicator of Alzheimer’s disease. *Int J Environ Res Public Health.* (2021) 18:4211. 10.3390/ijerph18084211 33921182 PMC8071516

[51] Ihara Y Takeshita T Kageyama S Matsumi R Asakawa M Shibata Y Identification of initial colonizing bacteria in dental plaques from young adults using full-length 16s rRNA gene sequencing. *mSystems.* (2019) 4:e00360–19. 10.1128/mSystems.00360-19 31481603 PMC6722423

