# Supplementary material for: Xylitol-Containing Chewing Gum Reduces Cariogenic and Periodontopathic Bacteria in Dental Plaque—Microbiome Investigation
Source: Front Nutr. 2022 May 11;9:882636. doi: 10.3389/fnut.2022.882636 (PMC9131035; doi:10.3389/fnut.2022.882636)
Supplement: Supplementary file 1 [file Data_Sheet_1.PDF]

Table S1. The questionnaire for participants after joining two weeks experiments

Evaluation of the effect of xylitol chewing gum on plaque formation - Questionnaire (English version)

Serial number: ______________________

1. Do you have a smoking habit? □YES □NO

2. Do you have severe dependence related to alcohol? □YES □NO

3. Do you have any systemic diseases such as Connective Tissue Diseases, Systemic Lupus Erythematosus, ㄇ Autoimmune Disease?

____

4. Have you taken any medication in the past 2 weeks?

□ YES Can you tell us what kind of medicine is it?___________________________

□ NO

5. In the past two weeks during this experimental period, did you use chewing gum regularly as directed by researchers?

□ YES Frequently (4~5 times/per day) □Sometimes (2~3 times/per day) □Seldom (1 time/per day) □Not applicable

6. In the past two weeks during this experimental period, did you use chewing gum at least 5 minutes each time?

□YES (at least 5 minutes for each chewing) □Occasionally (Approximately 3~5 minutes) □Rarely (Less than 2 minutes) □Not applicable

7. At baseline (M0) and effect examination (M1), did you **not** brush your teeth at least 48 hours before the examination?

□Yes □Only baseline examination □Only effect examination □Neither

8. In the past two weeks, did you use the toothpaste we prepared for you?

□Yes □Only baseline examination □Only effect examination □Neither

9. At baseline (M0) and effect examination (M1), did you **not** eat at least 6 hours and **not** drink at least 2 hours?

□Yes □Only baseline examination □Only effect examination □Neither

10. Do you have any feedback on this chewing gum product？

□YES____________________   □NO   □Not applicable
