# Supplementary material for: Xylitol-Containing Chewing Gum Reduces Cariogenic and Periodontopathic Bacteria in Dental Plaque—Microbiome Investigation
Source: Front Nutr. 2022 May 11;9:882636. doi: 10.3389/fnut.2022.882636 (PMC9131035; doi:10.3389/fnut.2022.882636)
Supplement: Supplementary file 2 [file Data_Sheet_2.PDF]

Table S2. The demographics and specific information of study participants

Serial number	Age	Gender	Number of teeth	DT value	MT value	FT value	DMFT	M0_Dental Plaque weight (mg)	M1_Dental Plaque weight (mg)	M0_pH value	M1_pH value
C1	22	F	28	2	4	4	10	32	25.9	6.44	6.49
C2	23	F	30	1	2	6	9	73.4	68.2	6.53	6.5
C3	22	M	29	0	3	8	11	63.3	40.4	6.45	6.86
C4	24	F	29	3	3	3	9	107.7	49.5	6.74	6.7
C5	23	F	30	0	2	10	12	69.9	34.3	5	6.36
C6	23	M	28	2	4	1	7	19.2	22.1	6.96	6.99
C7	24	M	30	1	2	14	17	13.8	57.9	6.71	6.58
C8	31	M	28	4	4	11	19	36.4	15.1	7.12	5.27
C9	34	M	30	2	2	0	4	13.2	14	5.49	6.8
C10	25	F	29	0	3	1	4	20.3	35.5	6.83	6.49
C11	23	M	28	7	4	3	14	19.1	22.1	6.25	6.8
X1	24	F	28	1	4	1	6	41.8	13	6.51	7.34
X2	23	F	30	1	2	5	8	59.5	41.9	6.79	6.58
X3	22	M	30	0	2	0	2	106.1	53	6.91	6.41
X4	33	M	28	4	4	6	14	17.2	15.1	7.16	6.97
X5	23	M	32	7	0	3	10	57.6	26	6.52	5.67
X6	22	F	28	1	4	0	5	11.1	16.7	7.34	6.81
X7	21	F	32	1	0	1	2	34.8	34.9	5.37	6.01
X8	20	F	28	6	4	3	13	30.5	18.6	6.7	6.3
X9	23	M	28	2	4	6	12	31.7	26.4	6.2	6.65
X10	22	F	32	9	0	4	13	38	37.8	6.56	6.61
X11	24	M	31	4	1	2	7	60.1	54.3	6.55	5.64
X12	23	M	26	0	6	3	9	32.5	30	6.28	6.34
X13	23	F	26	1	6	8	15	22.4	14.7	4.59	4.48
