# Supplementary material for: Xylitol-Containing Chewing Gum Reduces Cariogenic and Periodontopathic Bacteria in Dental Plaque—Microbiome Investigation
Source: Front Nutr. 2022 May 11;9:882636. doi: 10.3389/fnut.2022.882636 (PMC9131035; doi:10.3389/fnut.2022.882636)
Supplement: Supplementary file 3 [file Data_Sheet_3.PDF]

Table S3. The number of unique tags, Good's coverage, and alpha diversity estimates of oral bacterial communities

**Variable**	**Control**		**Xylitol gum**	
	**M0**	**M1**	**M0**	**M1**
Unique tags	11341 ± 2027	11422 ± 2852	10823 ± 1788	11304 ± 1334
Good's coverage	0.990 ± 0.002	0.990 ± 0.002	0.990 ± 0.002	0.990 ± 0.002
Chao1 (Richness index)	178.56 ± 25.07	176.48 ± 26.12	187.26 ± 34.59	181.01 ± 29.73
Shannon (Diversity index)	5.10 ± 0.55	4.75 ± 0.43	5.20 ± 0.58	4.69* ± 0.74

* $p < 0.05$ by two-tailed paired t-test; The oral microbial diversity were significantly decreased following chewing xylitol gum treatment.
